# IgA Dysfunction Induced by Early-Lifetime Low-Dose Antibiotics Exposure Aggravates Diet–Induced Metabolic Syndrome

**DOI:** 10.3390/antibiotics14060574

**Published:** 2025-06-03

**Authors:** Xue Han, Yue Qin, Jielong Guo, Weidong Huang, Yilin You, Jicheng Zhan, Yue Yin

**Affiliations:** 1 State Key Laboratory of Vascular Homeostasis and Remodeling, Department of Pharmacology, School of Basic Medical Sciences, Peking University, Beijing 100191, China; hanxuehx313@bjmu.edu.cn; 2 Beijing Key Laboratory of Viticulture and Enology, College of Food Science and Nutritional Engineering, China Agricultural University, Beijing 100083, Chinayilinyou@cau.edu.cn (Y.Y.)

**Keywords:** low-dose penicillin, IgA, gut microbiota, metabolic syndrome

## Abstract

**Background:** Low-dose antibiotic contamination in animal feed is a persistent global food safety challenge. Transient early-life exposure to low-dose penicillin (LDP) is known to induce metabolic syndrome (MetS) in adult mice, but the underlying mechanisms are unclear. **Introduction:** This study investigated the role of gut microbiota (GM) and intestinal immunity in mediating the long-term metabolic effects of early-life LDP exposure. **Methods:** Mice were exposed to LDP transiently during early life. GM composition was analyzed. Intestinal IgA responses were quantified. Bacterial encroachment, systemic and adipose tissue inflammation, and diet-induced MetS were assessed. Germ-free (GF) mice received GM transplants from LDP-exposed or control mice to test causality and persistence. **Results:** Early-life LDP exposure significantly disrupted GM composition, particularly in the ileum, in 30-day-old mice. These GM alterations caused persistent suppression of intestinal IgA responses, evidenced by reduced IgA-producing cells and sIgA levels. This suppression was constrained to early-life exposure: transferring LDP-modified GM to GF mice produced only a transient reduction in fecal sIgA. The LDP-induced sIgA reduction decreased IgA binding of bacteria, leading to increased bacterial encroachment and systemic and adipose tissue inflammation. These pathological changes exacerbated diet-induced MetS. **Discussion:** Our findings demonstrate that early-life LDP exposure induces persistent intestinal IgA deficiency through lasting GM alterations initiated in early development. This deficiency drives bacterial encroachment, inflammation, and ultimately exacerbates MetS. **Conclusions:** The exacerbation of diet-induced metabolic syndrome by early-life LDP exposure occurs through an intestinal sIgA-dependent pathway triggered by persistent GM disruption. This highlights a critical mechanism linking early-life antibiotic exposure, gut immune dysfunction, and long-term metabolic health, with significant implications for food safety.

## 1. Introduction

The early-lifetime period is critical for the development of immunity and metabolism, since the correct colonization and maturation of GM are delicately controlled by the host, and have a lifelong impact on the health of the host [1,2,3,4,5]. The disruption of the neonatal microbiome can result in lifelong changes in the GM composition and has been linked to various conditions such as obesity, asthma, and inflammatory bowel disease (IBD) [6,7,8,9]. A main factor disturbing the establishment and maturation of the early GM is the exposure to antibiotics through treatment (high dose) or unnoticed subtherapeutic exposure to low-dose antibiotics in contaminated animal foods (for example, meat, eggs, milk, and aquatic products) [2].

The overuse of antibiotics in young children for medical purposes in the US and China is quite serious [2,10], and efforts have been made in recent years by the government to reduce the therapeutic usage of antibiotics [11]. However, in contrast to therapeutic usage of antibiotics, low-dose antibiotic exposure via contaminated animal food is more inconspicuous and harder to avoid [1,4]. In the USA, an estimated number of 17 million kg of antibiotics were used in farm animals as compared to an estimated 4 million kg in humans per year, and a majority of these antibiotics are given in subtherapeutic doses to healthy chicken, cattle, and pigs to promote weight gain [12].

Penicillin is still one of the main antibiotics being used for animals and it is detected in contaminated food [2,13]. Previous studies have shown that short-term low-dose penicillin (LDP) treatment during early lifetime transiently disturbed the fecal microbiota and resulted in metabolic syndrome (MetS) in mice [1,14]. However, the underlying mechanisms and differences in microbial composition between intestinal luminal and mucosal samples were still largely unknown [1].

It has been previously reported that transient high-dose antibiotics treatment during early life resulted in a relatively long-lasting (up to 70 d of age) reduction in fecal sIgA [15,16], which is the most abundant immunoglobulin isotype in humans and mice and is a master controller of GM that has a key role in regulating MetS [17]. However, the prolonged influence of high-dose antibiotics on the intestinal sIgA and its correlation with metabolic disease was not determined [15,16].

We aimed to investigate whether the interaction between GM and intestinal IgA response plays a role in mediating the LDP-induced MetS. By using this LDP-induced MetS mouse model [1], we found that transient LDP treatment persistently reduced fecal sIgA and intestinal IgA^+^ B cells. We also found that this persistent influence of LDP on IgA response was both GM- and early life-dependent. Moreover, using sIgA-deficient (*Pigr*^−/−^) mice, we demonstrated that transient LDP treatment increased the ileal bacterial encroachment and translocation, leading to the exaggerated diet-induced MetS in WT mice in a sIgA-dependent manner.

## 2. Results

### 2.1. Early-Life LDP Disrupts Gut Microbiota

**Figure 1:**
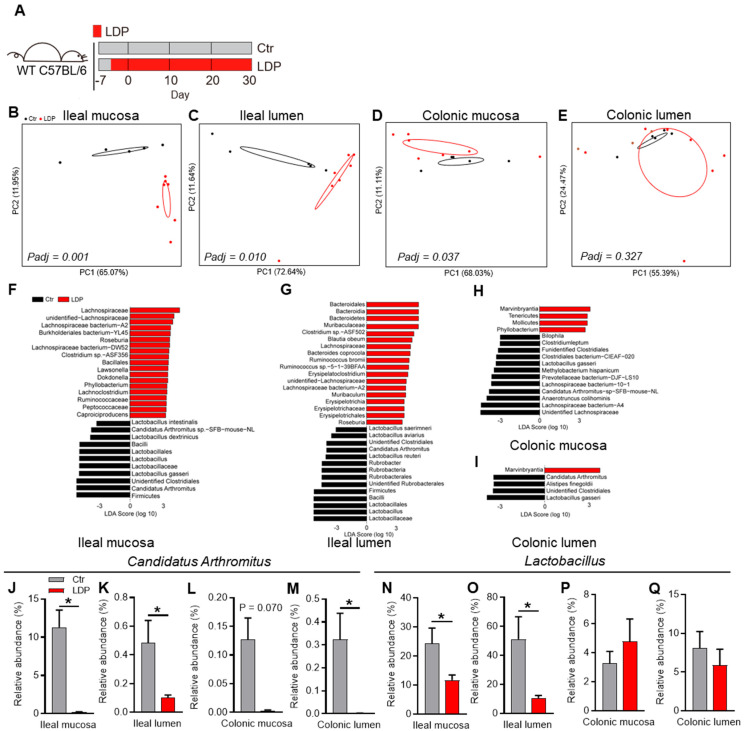
Early-life LDP disrupt gut microbiota. (**A**) Study design: Nine-week-old wild-type (WT) female and male C57BL/6 mice were randomly paired (1:1) and co-housed for 4 d (one estrous cycle for house mouse). After co-housing, females were separated from males and fed solely, and pregnancies and due dates were speculated according to the body weight changes. Several days before birth, pups were exposed to LDP, as previously described (10 mg/L, ~1.5 mg/kg body weight) through their mothers or drinking water until 30 d of age (LDP) or not (Ctr). The mice in each group came from at least three dams and were housed in at least two cages (*n* = 2–3 per cage) to avoid the cage-effect. Pups were separated from their mothers at 21 d of age and female and male pups were housed together until 30 d. (**B**–**E**) The principal coordinate analysis (PCoA) based on the weighted UniFrac distance indicated the differences in the bacterial composition of ileal mucosal (**B**), ileal lumen (**C**), colonic mucosa (**D**), and colonic lumen (**E**) samples, *n* = 5 for the Ctr mice and *n* = 7 for the LDP mice. Significance was determined using the Adonis test. (**F**–**I**) Discrepant bacterial species between the LDP and the Ctr mice were identified by LEfSe analysis, *n* = 5 for the Ctr mice and *n* = 7 for the LDP mice. (**J**–**Q**) The relative abundance of *Candidatus Arthromitus* (**J**–**M**) and *Lactobacillus* (**N**–**Q**) from the different intestinal regions, as determined by 16S rRNA gene sequencing, *n* = 5 for the Ctr mice and *n* = 7 for the LDP mice. Data are presented as mean ± SEM. Statistical significance is determined by Mann–Whitney U test (* *p* < 0.05).

As female and male pups were housed together during early life, their GM composition was analyzed together here. Quantitative PCR (qPCR) using 16S universal or ITS1 primers [18] (Table S1) showed no significant differences in the bacterial counts or fungal loads of the intestinal samples, suggesting that the overall microbial loads were not influenced by LDP treatment (Figure S1A,B). However, LPD significantly influenced the bacterial compositions of the ileal mucosa (*Padj* = 0.001, Adonis test), ileal lumen (*Padj* = 0.010, Adonis test), and colonic mucosa (*Padj* = 0.037, Adonis test) samples (Figure 1B–D). Meanwhile, the bacterial composition of the colonic lumen was not significantly influenced (*Padj* = 0.327, Adonis test) (Figure 1E).

We then analyzed the influence of LDP on OTUs based on Linear Discriminant Analysis Effect Size (LEfSe). LDP treatment mainly resulted in a reduction in *Lactobacillus* and *Candidatus Arthromitus* (segmented filamentous bacteria (SFB)) in all examined intestinal regions (Figure 1F–Q). It is also worth noting that a broader difference was observed between the ileal samples of the LDP and Ctr mice than in colonic samples (Figure 1F–I).

In conclusion, LDP disturbed the intestinal bacterial composition, mainly significant reductions in SFB and *Lactobacillus*.

### 2.2. Early Life LDP Persistently Dampens Intestinal IgA Responses

**Figure 2:**
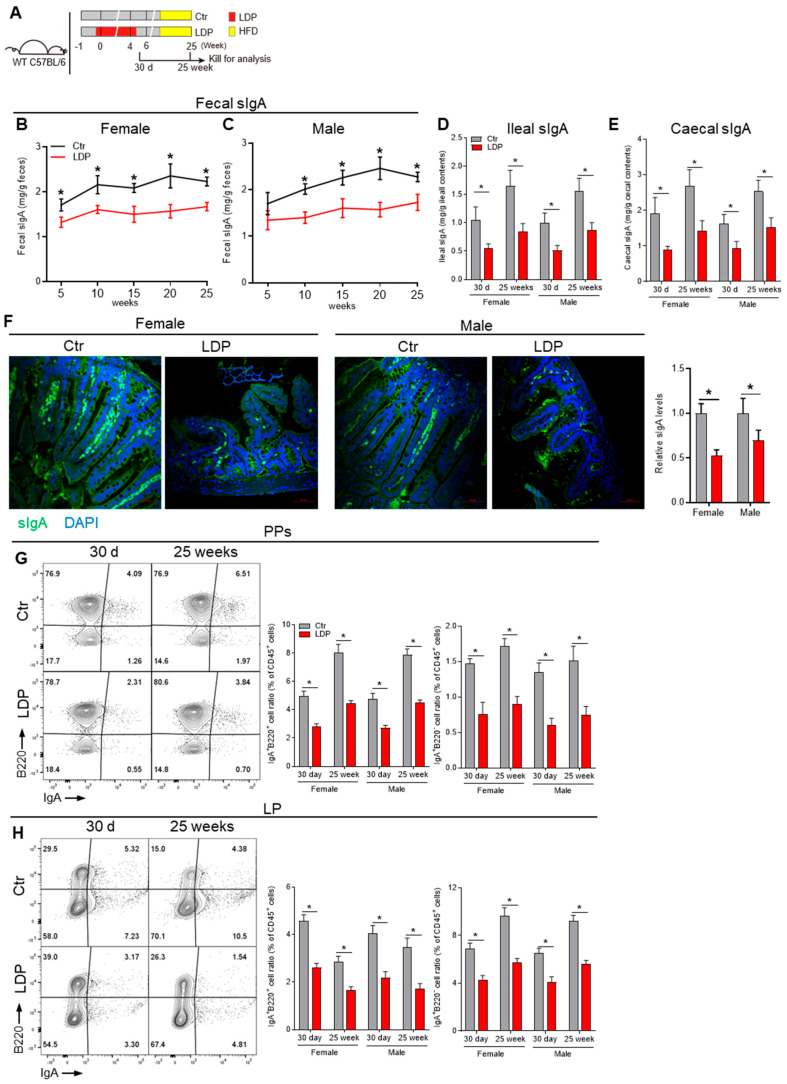
Early-life LDP persistently dampens intestinal IgA responses. (**A**) Study design: LDP treatment during early life was the same as in Figure 1A, but the experimental period was extended to 25 weeks, and a Western diet (45% energy from lard) started at 6 weeks was used to accelerate the metabolic effects of LDP. At 30 d and 25 weeks of age, mice were killed for the analysis of IgA response. (**B**,**C**) Changes in fecal SIgA levels, *n* = 5. (**D**,**E**) Ileal (**D**) and caecal (**E**) sIgA levels in 30 d-old and 25-week-old mice, *n* = 5. (**F**) Representing immunofluorescence images showing the sIgA levels in distal ileum of 30 d mice, *n* = 4. (**G**,**H**) Frequency of IgA-producing B cells (IgA^+^B220^+^) and plasma cells (IgA^+^B220^−^) within the Peyer’s patches (PPs) (**G**) and distal small intestine lamina propira (LP) (**H**), *n* = 5. Data are presented as mean ± SEM. Statistical significance is determined by Mann–Whitney U test (* *p* < 0.05).

As the intestinal IgA response is closely related to the GM composition and development of MetS [17], we sought to determine the influence of early-life LDP on the intestinal IgA response. There was no significant difference in the fecal sIgA among all 14-day and 21-day mice, suggesting that the passive sIgA received from their mothers via breast milk was not influenced by LDP (Figure S2A). In 30 d male and female pups, when mice were able to actively generate intestinal sIgA [6], LDP decreased the sIgA levels in ileal and caecal contents (Figure 2D,E).

We then determined the long-term (25 weeks) effects of LDP on intestinal IgA response. As a Western diet (45% energy from lard) has been shown to accelerate the metabolic effects of LDP [1], the diet of mice was changed to a Western diet at 6 weeks of age (Figure 2A). LDP reduced fecal sIgA levels throughout the 25-week experiment (Figure 2B,C). The ileal and caecal sIgA levels in 25-week mice were also significantly decreased by LDP treatment (Figure 2D,E). Immunofluorescence analysis of sIgA in the ileum further verified these results (Figure 2F). The serum IgA levels showed no significant changes (Figure S2B). 

Intestinal sIgA is mainly produced by IgA^+^ plasma cells (PCs) in the lamina propria (LP) [17]. LDP significantly decreased IgA^+^B220^+^ B cells and IgA^+^B220^−^ PCs in the LP of both 30 d and 25-week mice (Figure 2H). IgA^+^ PCs mainly originated from Peyer’s patches (PPs) [17]. Consistently, LDP significantly decreased IgA^+^B220^+^ B cells and IgA+B220^−^ PCs in the PPs of both 30 d and 25-week mice (Figure 2G).

In total, early-life LDP persistently dampened intestinal IgA response and reduced sIgA production.

### 2.3. LDP-Induced Inhibition of IgA Response Is GM- and Early Life-Dependent

As the intestinal IgA response is greatly dependent on the colonization and composition of GM [19,20], we sought to determine the correlation between LDP-induced changes in GM and inhibition of the intestinal IgA response.

**Figure 3:**
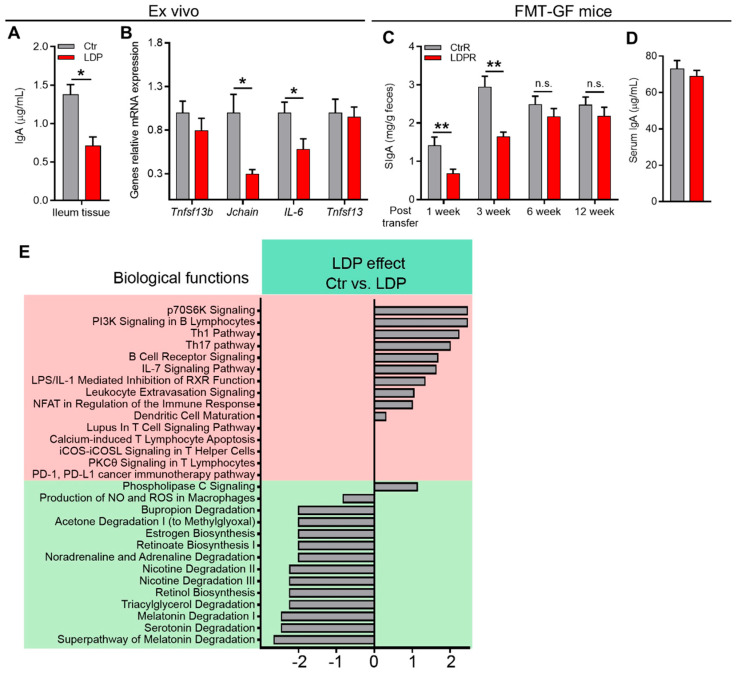
LDP-induced inhibition of IgA response is GM- and early life-dependent. (**A**,**B**) IgA production (**A**) and relative gene expression (**B**) in the mouse ileum during ex vivo cultivation with fecal microbiota antigens derived from the Ctr and LDP mice, *n* = 4. (**C**,**D**) The fecal (**C**) and serum (**D**) (s)IgA levels of the conventionalized GF mice transferred fecal microbiota from 30 d Ctr and LDP mice, *n* = 8. (**E**) Predicted canonical pathways determined by Ingenuity Pathway Analysis of the RNA-sequencing of ileal gene expression, *n* = 5. Data are presented as mean ± SEM. Statistical significance is determined by Mann–Whitney U test (* *p* < 0.05, ** *p* < 0.01, n.s. stands for not significant (*p*> 0.05)).

We first examined the ex vivo IgA-inducing capabilities of the antigens derived from the feces of 21 d mice, as previously described [7,21]. Briefly, antigens separated from the feces through centrifugation were normalized and co-cultured with ileum tissue samples obtained from 8-week-old SPF C57/BL6 mice to study the effects of these antigens on ileal IgA production. After 2 d of cultivation, the ileum tissue produced significantly more IgA when co-cultured with antigens derived from the feces of Ctr mice than from LDP mice (Figure 3A). Moreover, the mRNA expression of the Jchain and IL-6 of the ileum tissue was also higher, while tumor necrosis factor (ligand) superfamily, member 13 (also known as APRIL) and 13b (also known as BAFF) were not influenced by antigens from LDP mice (Figure 3B), suggesting that Ctr antigens may increase IgA production through enhancing the survival and activity of IgA^+^ PCs [17].

These results suggest that LDP inhibits the intestinal immune response to GM RNA-sequencing of the ileum from 30 d mice confirmed these results. Several biological functions related to IgA production and immune response to microbes were downregulated by LDP treatment, including PI3K signaling in B lymphocytes, B cell receptor signaling, dendritic cell maturation, and retinoate biosynthesis (Figure 3E).

To further determine the correlation between LDP-induced changes in GM and sIgA production, we transferred fecal microbiota from 30 d mice to age-matched germ-free (GF) mice and measured the fecal sIgA levels in recipients. One week post-transfer, LDP recipients (LDPR) exhibited significantly lower fecal sIgA levels compared to those of Ctr recipients (CtrR) (Figure 3C). The difference in the fecal SIgA levels lasted for 3 weeks after the transfer (Figure 3D). However, contrary to the donors, the fecal sIgA differences disappeared 6 weeks after the transfer (Figure 3C), suggesting that the time factor (early life) plays an important role in the prolonged influence of LDP-induced inhibition of IgA responses. Moreover, no significant difference in serum IgA 12 weeks after transfer was observed (Figure 3D).

Together, these results indicate that LDP-induced changes in GM mediated the inhibition of intestinal IgA response and that early life is critical for the long-lasting effects of LDP on intestinal IgA response.

### 2.4. LDP Promotes Systemic and Adipose Inflammation Through Enhancing Bacterial Encroachment

Intestinal SIgA can bind to the microbial antigens, preventing bacterial encroachment and adhesion to intestinal epithelial cells (IECs) as well as the translocation of bacteria and their metabolites, such as lipopolysaccharides (LPS) and flagellin, therefore protecting hosts from inflammation [17]. We therefore sought to investigate the influences of LDP-induced reduction in intestinal sIgA on bacterial encroachment and inflammation.

**Figure 4:**
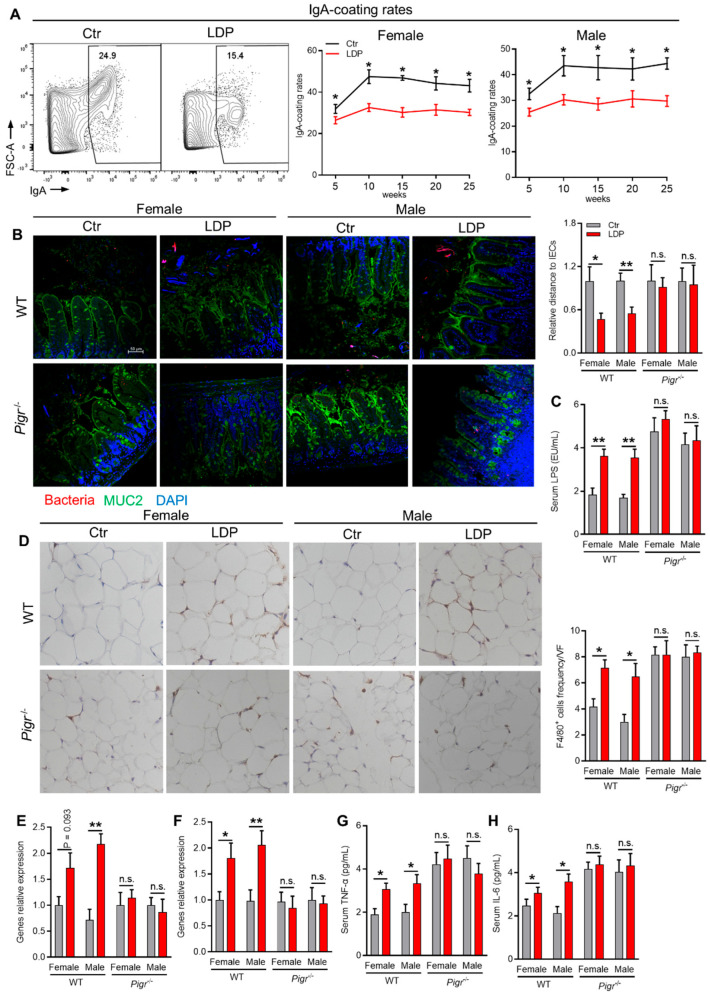
LDP promotes systemic and adipose inflammation through enhancing bacterial encroachment. (**A**) Flow cytometric analysis showing the changes in IgA-coating rates of fecal microbiota, n = 5. (**B**) Representative images of fluorescence in situ hybridization analysis targeting bacteria in ileum (left) and statistical analysis of the relative distance of bacteria to intestinal epithelial cells. MUC 2, green; bacteria, red; and DNA, blue; n = 5 for *Pigr*^−/−^ mice, n = 8 for WT Ctr and n = 9 for WT LDP mice. Distances of bacteria that translocate into the villi are calculated as zero. (**C**) Serum lipopolysaccharides concentration; n = 5 for *Pigr*^−/−^ mice, n = 8 for WT Ctr and n = 9 for WT LDP mice. (**D**) Representative immunohistochemical analysis targeting F4/80 in visceral adipose tissue, n = 6. (**E**,**F**) Genes relative expression in adipose tissue, n = 5 for *Pigr*^−/−^ mice, n = 8 for WT Ctr and n = 9 for WT LDP mice. (**G**,**H**) Serum TNF-α (**G**) and IL-6 (**H**) levels, n = 5 for *Pigr*^−/−^ mice, n = 8 for WT Ctr and n = 9 for WT LDP mice. Data are presented as mean ± SEM. Statistical significance is determined by Mann–Whitney U test (* *p* < 0.05, ** *p* < 0.01, n.s. stands for not significant (*p*> 0.05)).

As expected, LDP significantly decreased the binding of GM by IgA across the 25-week experiment (Figure 4A). Consistent with this, fluorescence in situ hybridization (FISH) analysis targeting bacteria showed that LDP significantly reduced the distance between the bacteria and IECs in WT but not in sIgA-deficient (*Pigr*^−/−^) mice (Figure 4B). The deficiency of fecal sIgA in *Pigr*^−/−^ mice was verified by direct measurement of sIgA using ELISA (Figure S3). This LDP-induced compromised control of bacteria by sIgA was accompanied by an increase in serum LPS in WT but not *Pigr*^−/−^ mice (Figure 4C). Consistently, serum inflammatory factors, TNF-α and IL-6, were significantly increased in LDP-treated WT mice (Figure 4G,H).

We then determined the influence of LDP on adipose inflammation, a key factor involved in the development of insulin resistance [22]. A significant LDP-induced increase in macrophage infiltration in visceral adipose tissue was found in WT but not *Pigr*^−/−^ mice (Figure 4D). Gene expression analysis using qPCR also demonstrated LDP-induced increases in the mRNA expression of TNF-α and IL-6 in the visceral adipose tissue of WT mice (Figure 4E,F), suggesting enhanced adipose tissue inflammation in LDP-treated WT mice.

Taken together, LDP compromised sIgA-binding of GM, leading to enhanced bacterial encroachment and systemic and adipose tissue inflammation.

### 2.5. LDP Exaggerates Diet-Induced MetS in a sIgA-Dependent Manner

We then sought to determine whether LDP-induced inhibition of intestinal IgA response was involved in the LDP-exaggerated development of MetS.

**Figure 5:**
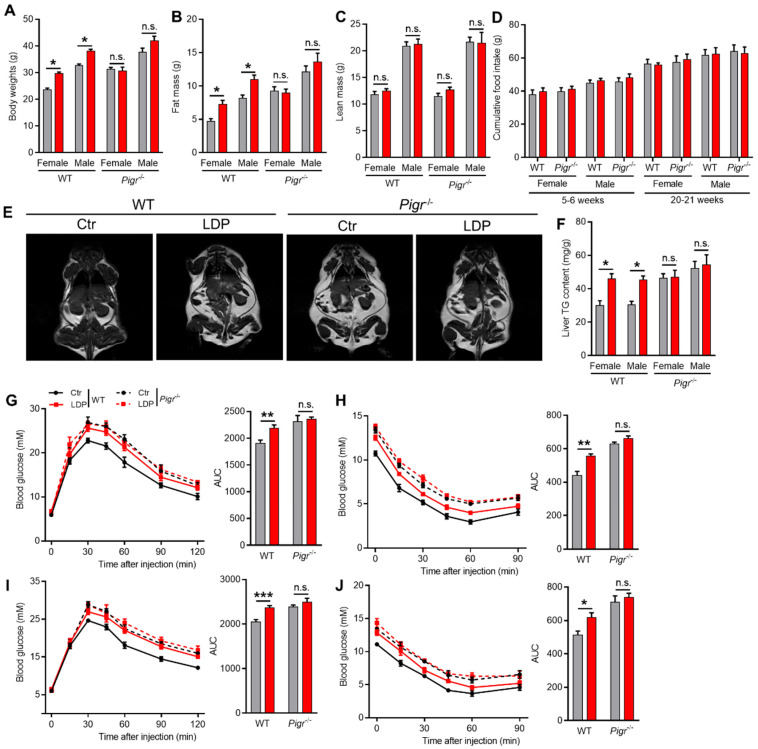
LDP exaggerates diet-induced MetS in sIgA-dependent manner. (**A**–**C**) Body (**A**), fat (**B**), and (**C**) fat masses, n = 5 for *Pigr*^−/−^ mice, n = 8 for WT Ctr and n = 9 for WT LDP mice. (**D**) Food intake, n = 5 for *Pigr*^−/−^ mice, n = 8 for WT Ctr and n = 9 for WT LDP mice. (**E**) Representative MRI images of the 25-week-old mice; the white areas represent lipids. (**F**) Hepatic triglyceride (TG) contents, n = 5 for *Pigr*^−/−^ mice, n = 8 for WT Ctr and n = 9 for WT LDP mice. (**G**–**J**) Blood glucose changes and the area under curve (AUC) during a GTT (**G**,**I**) or ITT (**H**,**J**) test, n = 5 for *Pigr*^−/−^ mice, n = 8 for WT Ctr and n = 9 for WT LDP mice. Data are presented as mean ± SEM. Statistical significance is determined by Mann–Whitney U test (* *p* < 0.05, ** *p* < 0.01, *** *p* < 0.001, n.s. stands for not significant (*p* > 0.05)).

Consistent with a previous study [1], LDP enhanced the development of Western diet-induced MetS in WT mice, including increases in body (Figure 5A) and (Figure 5B) fat masses, adiposity (Figure 5C), and hepatic triglyceride (TG) (Figure 5F). In addition, the glucose tolerance and insulin sensitivity were also impaired by LDP treatment in WT mice (Figure 5G–J). These changes were not related to the energy intake (Figure 5D). Notably, although *Pigr*^−/−^ mice showed exaggerated MetS compared to WT mice, no significant changes in the above parameters were observed between Ctr and LDP *Pigr*^−/−^ mice (Figure 5).

Collectively, LDP exacerbates diet-induced MetS through an sIgA-dependent pathway.

## 3. Discussion

In this study, we showed that the LDP treatment during early life disturbed GM composition, resulting in a persistent inhibition of the intestinal IgA response and a decrease in intestinal sIgA, which led to a disruption in the ileal bacterial composition, increases in bacterial encroachment and adipose inflammation, and an enhancement of diet-induced MetS.

The intestinal IgA^+^ B cell repertoire can be greatly shaped by the routine of GM colonization in GF mice [23]. Although this has not been verified in the newborn mice, considering their similarities (both firstly experience a GM-induced intestinal IgA^+^ B cells differentiation and maturation), similar mechanisms may also be the same in newborns. This influence on IgA repertoire can last for a long time, which may be partially attributed to the long-lived property of IgA^+^ B cells [24,25]. In line with this, we found that changes in GM during early life led to a persistent inhibition of intestinal IgA response. In addition, our results also demonstrated that IgA^+^ B cells differentiation in newborn mice is more sensitive to GM alteration than that in GF mice, since the intestinal SIgA reduction continued until the end of the experiment (25 weeks) in the WT SPF mice (whose GM was disturbed since birth) but disappeared in conventionalized GF mice (received GM at 30 d old) 6 weeks after the GM transfer.

A major role of intestinal SIgA is enhancing the clearance of microbes and preventing the encroachment and translocation of microbes and microbial antigens, therefore protecting the hosts from microbe-induced inflammation [17]. In accord, we found that the transient LDP-induced dampening of intestinal IgA response increased bacterial encroachment and translocation and serum LPS, which was accompanied by an exacerbation of adipose inflammation. A compromise of the intestinal IgA response has also been reported in high-fat diet (HFD)-induced obese mice [26]. In addition, deficiency in IgA (*Igha*^−/−^) exacerbates the HFD-induced adipose inflammation and MetS in mice [26] while an enhancement of IgA response through flagellin immunization can prevent HFD-induced MetS in a B and CD4^+^ T cells-dependent manner [27]. However, as both systemic (serum IgA) and mucosal sIgA were lacking in *Igha*^−/−^ mice, it is not clear which kind of IgA plays a major role in regulating metabolism in these studies [26,27]. We found that although LDP exposure reduced both the serum IgA and intestinal sIgA in 30-day-old WT mice, only the intestinal sIgA was still affected by the LDP in 25-week-old WT mice. In addition, through using sIgA-selectively deficient mice (*Pigr*^−/−^), we demonstrated that only mucosal sIgA, especially the intestinal sIgA, had a fundamental role in mediating LDP-induced alteration in metabolism.

## 4. Methods

### 4.1. Animals

#### 4.1.1. Wild-Type Specific Pathogen Free Animals

Wild-type (WT) C56BL/6J mice purchased at 8 weeks of age (Vital River Laboratory Animal Technology. Co., Ltd., Beijing, China) were randomly paired (1:1) after adapting for one week and fed a standard diet (#12450B, Research Diets, New Brunswick, NJ, USA). Female and male mice were co-housed at a 1:1 ratio for 4 days (one estrous cycle for house mouse), after which the female mice were individually housed. During and after cohousing, pregnancies and due dates were monitored and calculated according to the body weight changes.

Pups (and their mothers) were randomly assigned to two groups: low-dose penicillin (LDP)-free control group (Ctr) that received no antibiotics and LDP-treated control group (LDP) that received antibiotics. Pups were separated from their mothers at 21 days. Pups in every group were from at least three dams. For the LDP groups, dams received antibiotics at a dose of 10 mg/L to deliver approximately 1.5 mg per kg body weight about one week prior to birth and were continuously maintained on penicillin. This specific dosage regimen was effective for inducing metabolic MetS phenotypes in offspring during later developmental stages. [1]. LDP pups were exposed to penicillin until 30 days of age either through their mother or through drinking water. After the LDP treatment, pups were divided by sexes and kept 2~3/cage. All pups were fed on a normal diet (#D12450B, Research Diets) but transferred to a Western diet (45% energy from lard, D12451, Research Diets) at 6 weeks of age and allowed ad libitum access to food and water.

#### 4.1.2. Germ-Free Animals

For the microbiota transfer to germ-free (GF) animals’ experiment, feces were collected from 30-day-old donors from each group and immediately placed in prereduced anaerobically sterilized PBS, homogenized under anaerobic conditions, settled with gravity for 2 min, and then the supernatant was transferred to even-aged GF C57BL/6 mice. After transfer, the conventionalized GF mice were housed in standard SPF conditions, and food and water were provided ad libitum.

#### 4.1.3. SIgA-Deficient Mice

*Pigr*^+/−^ males and females, which can produce normal sIgA and therefore have similar GM to WT mice [6], were used to generate sIgA-deficient pups. Specifically, 8-week-old *Pigr*^+/−^ females and males were mated as specified above, the genotypes of pups were identified at 10 days of age, and only *Pigr*^−/−^ female and WT mice pups were chosen for the following experiment, as specified above.

#### 4.1.4. Animal Management and Sampling

Mice were housed in standard specific pathogen-free (SPF) conditions (12/12 h light–dark cycle, humidity at 50 ± 15%, with a temperature of 22 ± 2 °C), and food and water were provided ad libitum. The food used in this study was sterilized using radiation (25.0 kGy). Food intake was recorded every week. Body weight was recorded weekly. At the end of the experimental period, the mice were fasted for 12 h, and plasma was collected by eyeball extirpation. The lumen contents of the distal ileum and proximal colon were collected by washing the lumen with sterilized PBS, and the mucosal samples were collected by scraping the intestinal wall with sterilized glass slides. Then, the samples were stored at −80 °C for microbial analysis. The contents of the cecum were collected and stored at −80 °C for the analysis of sIgA. The weights of the liver, inguinal white adipose tissue (iWAT), epididymal white adipose tissue (eWAT), and mesenteric white adipose tissue (mWAT) were measured. Tissues were preserved at −80 °C for gene expression analysis (for ileum, all fat and mesentery were removed, and Peyer’s patches were excised from the ileum), and the liver, iWAT, colon, and ileum were fixed using 4% paraformaldehyde and used for immunofluorescence analysis.

### 4.2. Ileum Tissue Culture

Mouse ileum was obtained and cultured as previously described with some modification [21]. Distal ileum samples containing no Peyer’s patches were washed and cultured using RPMI 1640 supplemented with 10% FCS and penicillin/streptomycin 50 mg/mL at 37 °C and 5% CO_2_ in 24-well plates. Two ileum samples per well (~3 mm) were cultured for each group (n = 4). Two days after co-culture with antigens, culture medium was collected and supernatants were centrifuged and stored at −80 °C for the analysis of IgA and cells were collected for the analysis gene expression. Four eight-week-old male SPF C57/BL6 mice were used for sampling. The ileum samples of each group were taken from all four mice and comprised the same intestinal regions.

### 4.3. Isolation and Analysis of Immune Cells from the Intestine

Lymphocytes from the intestinal lamina propira (LP) and Peyer’s Patches (PPs) were isolated by adapting a method previously described [26]. Distal ileum (~10 cm prior to the cecum) was extracted, removing all mesentery fat as possible and PPs, and collected in ice-cold harvest media (RPMI 1640 (Sigma, St. Louis, MO, USA) supplemented with 5% FBS (Gibco, Waltham, MA, USA), 15 mM HEPES, penicillin–streptomycin (Gibco), pH 7.4). Extracted intestines were cut open longitudinally into 2~3 mm pieces in the wash buffer. Bowel pieces were transferred to an EDTA-containing stripping buffer (Hank’s balanced salt solution (Gibco) supplemented with 2% FBS (Gibco), 1.3 mM EDTA, 15 mM HEPES, penicillin-streptomycin (Gibco), pH 7.4) and shaken vigorously at 37 °C for 20 min, and then vortexed gently for a few seconds. This step was repeated two times. Gut pieces were then washed in cold harvest medium to remove residual EDTA before transfer into a digestion buffer (RPMI 1640 supplemented with 10% FBS (Gibco), 10 mM sodium pyruvate, penicillin–streptomycin antibiotics (Gibco), 15 mM HEPES, collagenase type I (100 U/mL, Sigma), DNase I (0.5 mg/mL, Sangon, Shanghai, China), and 1 mM CaCl_2_ and MgCl_2_), where it was minced finely with scissors, followed by a 45 min incubation at 37 °C with shaking. The resulting suspension of LP immune cells collected from the previous step were filtered twice through a 100- and 40-μmnylon cell strainer to obtain a single-cell suspension. PPs were mechanically disrupted in the harvest medium, and the homogenate was filtered through a 70 μm cell strainer to obtain a single-cell suspension. Single cells were stimulated with 50 ng/mL phorbolmyristate acetate and 1 μM Ionomycin in the presence of 5 μg/mL Brefeldin A. Following this in vitro stimulation, cells were stained with Fixable Viability Stain 780 (BD), anti-CD45.2, anti-CD4, anti-CD19, anti-CD45R, and anti-Tcrβ before being fixed and permeabilized using Transcription Factor Buffer (BD Pharmingen, San Diego, CA, USA). After fixation and permeabilization, cells were further stained with anti-IgA, anti-Foxp3, and anti-RORγ amtibodies. Cells were acquired on an LSRFortessa^TM^ Cell Analyzer (BD) and analyzed with FlowJo V10 (Tree Star, Ashland, OR, USA) software.

### 4.4. Quantitative Real-Time PCR (qPCR) Analysis

Total RNA was extracted using TRIzol^TM^ reagent (Invitrogen, Waltham, MA, USA) according to the manufacturer’s instructions. Reverse transcription of the total RNA (2.5 μg) was performed with a high-capacity cDNA reverse transcription kit (Promega Biotech Co., Ltd., Madison, WI, USA). qPCR was run in triplicate for each sample and analyzed in a LightCycler 480 real-time PCR system (Roche, Basel, Switzerland). Data were normalized to the internal control β-actin and analyzed using the ΔΔCT method. The expression of genes in iWAT, the liver, ileum and colon, as well as the bacterial and fungal load, were determined through qPCR (the related genes and primers used are shown in Table S1).

Quantification of the bacterial and fungal loads through qPCR was conducted as previously described [28]. Briefly, the total bacterial DNA was isolated from the samples with a QIAamp DNA Stool Mini Kit (Qiagen, Hilden, Germany) following the manufacturer’s instructions. For the isolation of fungal DNA, samples were suspended in 50 mM Tris buffer (pH 7.5) supplemented with 1 mM EDTA, 0.2% b-mercaptoethanol and 1000 units/mL of lyticase (Sigma), incubated at 37 °C for 30 min to disrupt fungal cells as described [29], prior to processing through the QIAamp DNA Stool Mini Kit (Qiagen). The DNA was then subjected to qPCR using a QuantiFast SYBR Green PCR kit (Bio-Rad, Hercules, CA, USA) with specific primers (Table S1).

### 4.5. Determination of Body Composition Through MRI

MRI experiments were performed on 25-week-old mice. The body composition was determined using MesoQMR instrument (Testniumag, Shanghai, China) with a 60 mm receiver and 0.5 ± 0.08 T magnetic field strength. To obtain high resolution scanned MRI images, MRI measurements were performed on a 7.0 T Varian MRI instrument (Varian Medical Systems, Palo Alto, CA, USA) using a 40 mm volume and receiver coil at the Institute of Laboratory Animal Sciences, Chinese Academy of Medical Sciences. Prior to the experiments, the mice were initially anesthetized with 2% isoflurane in a dedicated chamber. During the course of MRI, anesthesia levels were reduced to 1.5–1% in a combination of medical air and medical oxygen. The mice were positioned in the prone position, and respiratory-gated image acquisition was performed. MRI images of the mice were analyzed by Argus software v5.0.

### 4.6. Quantification of SIgA

Intestinal/fecal SIgA levels were determined using ELISA kits (Sigma-Aldrich, St. Louis, MO, USA) according to the manufacturer’s recommendations.

Separation of antigens and determination of IgA to the specific antigens were conducted as described with some modification [7,27]. Briefly, to prepare antigens from cecal contents of mice, cecal contents of mice from each group were normalized by bacterial loads (determined by qPCR as previously described [18]), pooled (n = 3) in PBS (sterilized with a 0.22 μm filter), vortexed for 5 min, and centrifuged for 5 min at 13,000 RPM, 4 °C.

### 4.7. Immunofluorescence and Immunohistochemistry

Tissue sections for immunohistochemical testing were prepared on poly-L-lysine-pretreated coverslips. Immunohistochemical staining was performed according to a standard protocol using antibodies against F4/80 at a 1:200 dilution. The samples were incubated overnight in a humidified chamber at 4 °C. Secondary antibodies for immunohistochemical staining were purchased from Invitrogen. All images were acquired on an Olympus BX51 system (Olympus, Tokyo, Japan) and processed using ImageJ software, version 1.8.0. Immunostaining for IgA was conducted using FITC-conjugated anti-IgA antibody at a 1:500 dilution. The samples were incubated overnight at 4 °C. Observations and analyses were performed with a Zeiss LSM 700 confocal microscope (Zeiss, Jena, Germany).

### 4.8. Localization of Bacteria by Fluorescent In Situ Hybridization

The localization of bacteria by fluorescent in situ hybridization (FISH) was conducted as previously described with some modifications [30]. Briefly, distal ileum (second cm from the caecum) containing fecal material was placed in methanol-Carnoy’s fixative solution (60% methanol, 30% chloroform, 10% glacial acetic acid) for a minimum of 3 h at room temperature. The hybridization step was performed at 50 °C overnight with an modified EUB338 probe [31] (EUB338-II, 5′-GCAGCCACCCGTAGGTGT-3′, with a 5′ Texas Red label) diluted to a final concentration of 10 mg/mL in hybridization buffer (20 mM Tris-HCl, pH7.4, 0.9 M NaCl, 0.1% SDS, 20% formamide). After washing, Mucin 2 primary antibody was diluted to 1:500 in a block solution and applied overnight at 4 °C. After washing, the block solution containing anti-rabbit FITC-conjugated secondary antibody diluted to 1:1000 was applied to the section for 2 h. After washing, slides were mounted using Prolong anti-fade mounting media (Life Technologies, Carlsbad, CA, USA). Observations were performed with a Zeiss LSM 700 confocal microscope with software Zen 2011 version 7.1. This software was used to determine the distance between bacteria and the epithelial cell monolayer.

### 4.9. Histology

Tissues fixed in 4% paraformaldehyde were cut into 5 μm sections after being embedded in paraffin. Multiple sections were prepared and stained with hematoxylin and eosin (H&E, Baton Rouge, LA, USA) for general morphological observation.

### 4.10. GM Analysis

The microbial community of fecal, mucosal, and lumen samples of colon and ileum were analyzed through the sequence of 16S rRNA gene V4 region. Briefly, total genome DNA from samples was extracted using the CTAB/SDS method. DNA concentration and purity was monitored on 1% agarose gels. According to the concentration, DNA was diluted to 1 ng/μL using sterile water. 16S rRNA gene V4 regions were amplified using a specific primer for the V4 region (515F-806R) with the barcode. All PCR reactions were carried out in 30 μL reactions with 15 μL of Phusion^®^ High-Fidelity PCR Master Mix (New England Biolabs, Ipswich, MA, USA); 0.2 μM of forward and reverse primers, and about 10 ng template DNA. Thermal cycling consisted of initial denaturation at 98 °C for 1 min, followed by 30 cycles of denaturation at 98 °C for 10 s, annealing at 50 °C for 30 s, and elongation at 72 °C for 30 s, and finally, at 72 °C for 5 min. The same volume of 1 × loading buffer (contained SYB green) was mixed with PCR products and electrophoresis was operated on 2% agarose gel for detection. PCR products were mixed in equidensity ratios. Then, a mixture of PCR products was purified with a GeneJETTM Gel Extraction Kit (Thermo Scientific, Waltham, MA, USA). Sequencing libraries were generated using Ion Plus Fragment Library Kit 48 rxns (Thermo Scientific) following the manufacturer’s recommendations. The library quality was assessed on the Qubit^®^ 2.0 Fluorometer (Thermo Scientific). Finally, the library was sequenced on an Ion S5TM XL platform and 400 bp/600 bp single-end reads were generated.

Single-end reads were assigned to samples based on their unique barcode and truncated by cutting off the barcode and primer sequence. Quality filtering on the raw reads was performed under specific filtering conditions to obtain the high-quality clean reads according to the Cutadapt (V1.9.1, http://cutadapt.readthedocs.io/en/stable/, accessed on 26 May 2025) quality-controlled process. The reads were compared with the reference database (Silva database, https://www.arb-silva.de/, accessed on 26 May 2025)) using UCHIME algorithm (UCHIME Algorithm, http://www.drive5.com/usearch/manual/uchime_algo.html, accessed on 26 May 2025)) to detect chimera sequences, and then the chimera sequences were removed [32]. Then, the clean reads were finally obtained.

Alpha diversity is applied in analyzing the complexity of species diversity for a sample through six indices, including Observed-species, Chao1, Shannon, Simpson, ACE, Good-coverage. All these indices in our samples were calculated with QIIME (V1.7.0) and displayed with R software (V2.15.3). Beta diversity analysis was used to evaluate differences in samples in species complexity; beta diversity on both weighted and unweighted unifrac was calculated by QIIME software (V1.7.0). Principal Coordinate Analysis (PCoA) was performed to obtain principal coordinates and visualize from complex, multidimensional data. A distance matrix of weighted or unweighted unifrac among samples obtained before was transformed into a new set of orthogonal axes, by which the maximum variation factor is demonstrated by first principal coordinate, and the second maximum one by the second principal coordinate, and so on. PCoA analysis was displayed by WGCNA package, stat packages, and the ggplot2 package in R software (V2.15.3).

### 4.11. RNA-Sequencing

Total RNA of the liver and ileum were extracted using TRIzol^TM^ reagent (Invitrogen) according to the manufacturer’s instructions. RNA degradation and contamination was monitored on 1% agarose gels; RNA purity was checked using the NanoPhotometer^®^ spectrophotometer (IMPLEN, Westlake Village, CA, USA); RNA concentration was measured using Qubit^®^ RNA Assay Kit in Qubit^®^ 2.0 Fluorometer (Life Technologies, Carlsbad, CA, USA); RNA integrity was assessed using the RNA Nano 6000 Assay Kit of the Bioanalyzer 2100 system (Agilent Technologies, Santa Clara, CA, USA). Then, a total amount of 3 μg RNA per sample was used as input material for the RNA sample preparations. Sequencing libraries were generated using NEBNext^®^ UltraTM RNA Library Prep Kit for Illumina^®^ (NEB, Ipswich, MA, USA) following the manufacturer’s recommendations and index codes were added to attribute sequences to each sample. Briefly, mRNA was purified from total RNA using poly-T oligo-attached magnetic beads. Fragmentation was carried out using divalent cations under elevated temperatures in NEBNext First Strand Synthesis Reaction Buffer (5X) (New England Biolabs, Ipswich, MA, USA). First strand cDNA was synthesized using random hexamer primer and M-MuLV Reverse Transcriptase (RNase H^−^). Second strand cDNA synthesis was subsequently performed using DNA Polymerase I and RNase H. Remaining overhangs were converted into blunt ends via exonuclease/polymerase activities. After adenylation of 3′ ends of DNA fragments, NEBNext Adaptors (New England Biolabs, Ipswich, MA, USA)with a hairpin loop structure were ligated to prepare for hybridization. In order to select cDNA fragments of preferentially 250~300 bp in length, the library fragments were purified with the AMPure XP system (Beckman Coulter, Beverly, MA, USA). Then, 3 μL USER Enzyme (NEB, USA) was used with size-selected, adaptor-ligated cDNA at 37 °C for 15 min followed by 5 min at 95 °C before PCR. Then, PCR was performed with Phusion High-Fidelity DNA polymerase, Universal PCR primers and Index (X) Primer. At last, PCR products were purified (AMPure XP system, Beckman Coulter, Beverly, MA, USA) and library quality was assessed on the Agilent Bioanalyzer 2100 system. The clustering of the index-coded samples was performed on a cBot Cluster Generation System using TruSeq PE Cluster Kit v3-cBot-HS (Illumia, San Diego, CA, USA) according to the manufacturer’s instructions. After cluster generation, the library preparations were sequenced on an Illumina Hiseq platform and 125 bp/150 bp paired-end reads were generated.

Raw data (raw reads) of fastq format were firstly processed through in-house perl scripts. In this step, clean data (clean reads) were obtained by removing reads containing adapters, reads containing ploy-N, and low-quality reads from raw data. At the same time, Q20, Q30, and GC content with the clean data were calculated. All the downstream analyses were based on the clean data with high quality. Reference genome and gene model annotation files were downloaded from the genome website directly. The index of the reference genome was built using Hisat2 (V2.0.5) and paired-end clean reads were aligned to the reference genome using Hisat2. We selected Hisat2 as the mapping tool because Hisat2 can generate a database of splice junctions based on the gene model annotation file and thus create a better mapping result than other non-splice mapping tools. featureCounts (V1.5.0-p3) was used to count the read numbers mapped to each gene., and then the FPKM of each gene was calculated based on the length of the gene and reads count mapped to this gene. FPKM, expected number of Fragments Per Kilobase of transcript sequence per Millions base pairs sequenced, considers the effect of sequencing depth and gene length for the reads count at the same time, and is currently the most commonly used method for estimating gene expression levels. Differential expression analysis of two conditions/groups (two biological replicates per condition) was performed using the DESeq2 R package (V1.16.1).

### 4.12. Statistical Analysis

All data reported in this paper are expressed as the means ± SEMs. Significant differences between the two groups were evaluated with the t-test. A *p* value of <0.05 was considered statistically significant. All statistics were analyzed by SPSS software 27, and all analyses were performed with GraphPad Prism 7.

## 5. Conclusions

Transient LDP exposure during early life disturbed the intestinal bacterial composition in mice, which led to a persistent dampening of the intestinal IgA response. Reduction in SIgA mediated the persistent influences of LDP on ileal microbiota, accompanied by increases in bacterial encroachment and translocation and adipose inflammation, enhancing the development of diet-induced MetS.

## Data Availability

The RNA-seq and 16S rRNA gene sequencing data supporting this research are available on NCBI with accession number PRJNA577425. All other data that support the findings of this study are available from the corresponding author upon reasonable request.

## Supplementary Materials

The following supporting information can be downloaded at: https://www.mdpi.com/article/10.3390/antibiotics14060574/s1, Figure S1. Bacterial and fungal loads, related to Figure 1. Figure S2. Serum IgA levels, related to Figure 2. Figure S3. Fecal sIgA levels, related to Figure 4. Figure S4. Gating strategy, related to all Figures. Table S1. Primers used in this study. Table S2. Key resources table.
