# Supplementary material for: IgA Dysfunction Induced by Early-Lifetime Low-Dose Antibiotics Exposure Aggravates Diet–Induced Metabolic Syndrome
Source: Antibiotics (Basel). 2025 Jun 3;14(6):574. doi: 10.3390/antibiotics14060574 (PMC12189073; doi:10.3390/antibiotics14060574)
Supplement: Supplementary file 1 [file antibiotics-14-00574-s001.zip › antibiotics-3628032-supplementary.pdf]

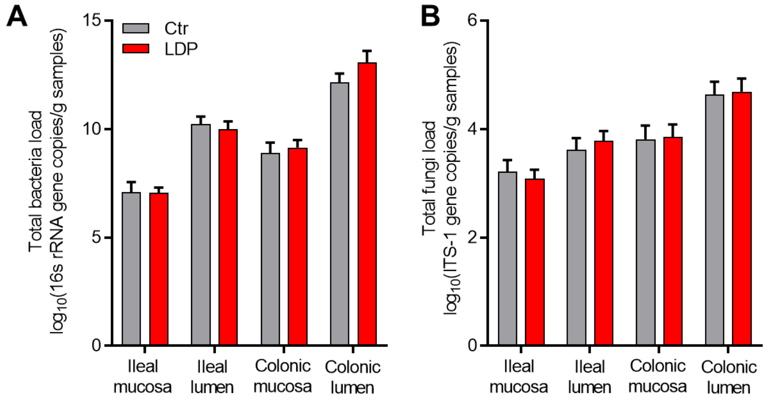

Figure S1. Bacterial and fungal loads, related to Figure 1. (**A** and **B**) Total bacterial (**A**) and fungal loads (**B**), as determined by qPCR, in the mucosal and lumimal samples of the ileum and colon, n = 8-9. Data presented as mean ± SEM. Statistical significance determined by Mann-Whitney U test.

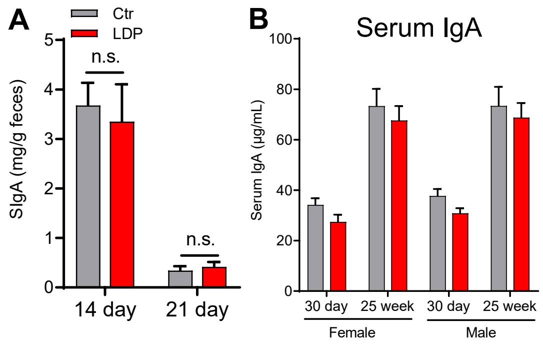

Figure S2. Serum IgA levels, related to Figure 2. (**A**) Fecal sIgA levels in pups, n = 9. (**B**) Serum IgA levels were determined by ELISA, n = 5. Data presented as mean ± SEM. Statistical significance determined by Mann-Whitney U test.

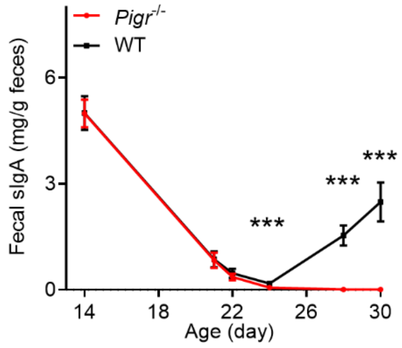

Figure S3. Fecal sIgA levels, related to Figure 4. Fecal sIgA changes in *Pigr*$^{-/-}$ and WT pups, n = 10. Data presented as mean ± SEM. Statistical significance determined by Mann-Whitney U test, ****P* < *0.001.*

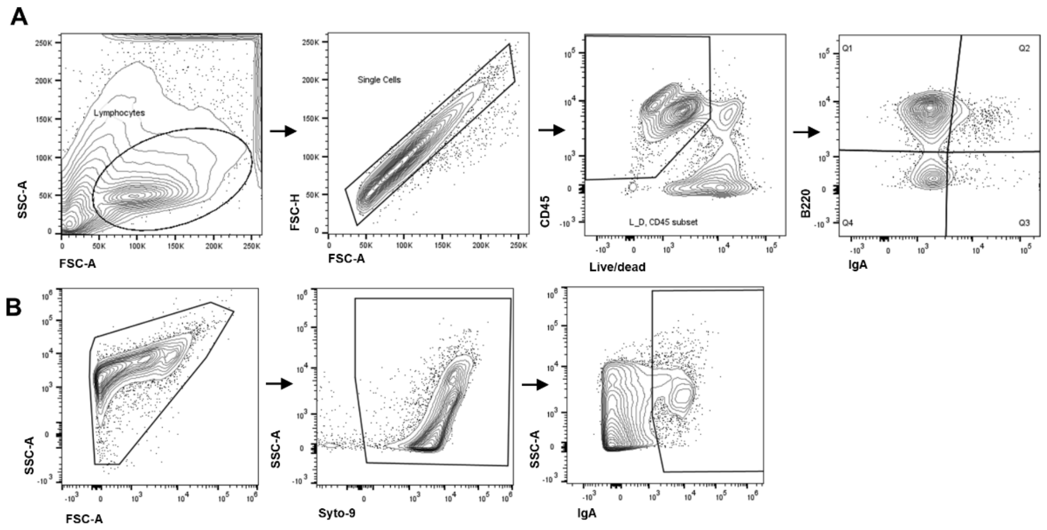

Figure S4. Gating strategy, related to all Figures. (**A**) IgA-producing cells: Lymphocytes, single cells, live CD45⁺ cells, IgA⁺B220⁺ cells (Q2), and IgA⁺B220⁻ cells (Q3). (**B**) IgA⁺ bacteria: small particles, bacteria, IgA⁺ bacteria.

Table S1. Primers used in this study.

GENE	FORWARD PRIMER (5'-3')	REVERSE PRIMER (5'-3')	SOURCE
Fungi ITS1	CTTGGTCATTTAGAGGAAGTAA	GCTGCGTTCTTCATCGAT	Sigma
Universal	AGAGTTTGATCCTGGCTCAG	GC	Sigma
FJCHAIN	TGACGACGAAGCGACCATTC	CTGCTGCCTCCCGTAGG	Sigma
β-actin	GGATGCAGAAGGAGATCACTG	AGT	Sigma
Pigr	AGTAACCGAGGCCTGTCCTT	TTCAAAGGGACAACAATT	Sigma
Tnfsf13b	TGATGGCTCTGATGGAATGA	CGGA	Sigma
IL-6	TAGTCCTTCCTACCCCAATTTC	CGATCCACACGGAGTACT	Sigma
Tnfsf13	C	TG	Sigma
	CTTTCGGTTGCTCTTTGGTTG	GTCACTCGGCAACTCAG	
		GA	
		AAAGCTTAGCAGCCACG	
		GTA	
		TTGGTCCTTAGCCACTCC	
		TTC	
		CGACAGCACAAGTCACA	
		GC	

Table S2. Key resources table.

Regent or Resources	Source	Identifier
Chemicals and ELISA kits		
Penicillin VK	Sigma	Cat# BP282

Mouse sIgA ELISA kit	Elabscience	Cat# E-EL-M1040
Zombie UV fixable viability kit	Biolegend	Cat# 423107
Deoxyribonuclease I	WBC	Cat# LS002145
Collagenase I	Sigma	Cat# C9263
Cell stimulation cocktail (containing protein	Invitrogen	Cat# 00-4975-93
transport inhibitor) (500×)	Biorad	Cat# 1708840
iScript™ Reverse Transcription Supermix for RT-qPCR	Biorad	Cat# 1725122
iTaq™ Universal SYBR® Green Supermix		
Antibody		
MUC2	Affinity	Cat#DF8390
Anti-mouse/human F4/80	Affinity	Cat# DF2789
IgA alpha chian (FITC)	Abcam	Cat#ab97234
Anti-mouse CD45.2-AF700	Biolegend	Cat# 109822
Anti-mouse B220-Bv510	Biolegend	Cat# 103247
Anti-mouse IgA-PE	Invitogen	Cat# 12-4204-83
